# Cutaneous tuberculosis with an unusual appearance and location

**DOI:** 10.11604/pamj.2019.32.81.18206

**Published:** 2019-02-18

**Authors:** Sqalli Houssaini Asmaa, Badreddine Hassam

**Affiliations:** 1 Dermatology and Venerology Department, Hospital IBN SINA, Faculty of Medicine and Pharmacy, Mohammed V University, Rabat, Morocco

**Keywords:** Tuberculosis, cutaneous tuberculosis, gums

## Image in medicine

Tuberculosis is a bacterial disease caused by mycobacterium tuberculosis. Its cutaneous form accounts for 2% of all extrapulmonary tuberculosis. We report a case of 30-year-old woman with nodules evolving for two years. Examination showed gums of genital and inguinocrural location, and scrofulous scars. Several diagnoses were discussed including tuberculosis, syphilis, actinomycosis, or cutaneous lymphoma. Skin biopsy revealed an epithelial giganto-cellular granuloma with caseating necrosis; the culture of a cutaneous fragment was positive for Mycobacterium Tuberculosis. Our case is original and unusual due to the uncommon clinical appearance and location of the nodules grouped in the inguinocrural region. The multiplicity of the clinical forms of cutaneous tuberculosis sometimes makes the diagnosis difficult. Gums and scrofulodermas remain the most frequent forms of cutaneous tuberculosis in Morocco. According to the immuno-anatomo-clinical classification, tuberculous gum is part of the multi-bacillary forms and is seen mostly in immunocompromised patients.

**Figure 1:**
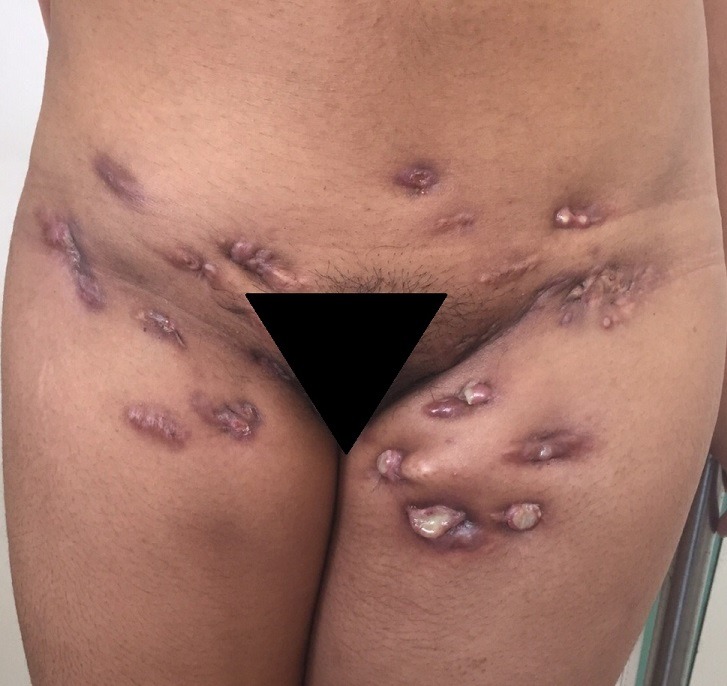
Gums in the inguinocrural region, scrofuloscars

